# 

**Published:** 1857-05

**Authors:** 


					﻿No. XII.]
MAY.
[Vol. XII.
BUFFALO
MEDICAL JOURNAL
AND
or
MEDICAL AND SURGICAL SCIENCE.
SANFORD B. HUNT, JI. D.,
ESXaXTOH.
BUFFALO, N. Y.:
THOMAS A LATHROPS’ STEAM PRESS.
Commercial Advertiser Buildings.
1857.
Price, $2.00 in advance, or $2.50 at the end of three months.
				

